# Aspirin for Venous Thromboembolism Prevention in Orthopaedic Surgery with Focus on Trauma and Arthroplasty: A Structured Evidence-Based Review of Randomised Trials, Guidelines, and Contemporary Practice Considerations

**DOI:** 10.3390/jcm15124550

**Published:** 2026-06-11

**Authors:** Christian Riediger, Mark Ferl, Maria Schönrogge

**Affiliations:** Department of Orthopaedics, University Hospital Magdeburg, Otto-von-Guericke-University Magdeburg, 39106 Magdeburg, Germany; mark.ferl@med.ovgu.de (M.F.); maria.schoenrogge@med.ovgu.de (M.S.)

**Keywords:** aspirin, venous thromboembolism, thromboprophylaxis, arthroplasty, trauma, surgery, randomised controlled trial

## Abstract

**Background:** Venous thromboembolism (VTE) remains a clinically relevant complication following major orthopaedic procedures, particularly total hip arthroplasty (THA), total knee arthroplasty (TKA), and fracture surgery. Although low-molecular-weight heparin (LMWH) and direct oral anticoagulants (DOACs) are widely regarded as standard pharmacological options, aspirin (acetylsalicylic acid, ASA) has gained renewed attention because of its low cost, oral administration, and favourable bleeding profile. However, the available evidence is heterogeneous, and its interpretation is complicated by differences in patient selection, timing and duration of prophylaxis, diagnostic methodology, aspirin dosing regimens, and the increasing adoption of modern fast-track arthroplasty pathways. **Methods:** A structured evidence-based review was conducted in accordance with PRISMA 2020 principles. PubMed, Embase, Web of Science, and the Cochrane Library were searched through September 2025 for randomised controlled trials (RCTs), major international clinical practice guidelines, and selected high-level studies relevant to the interpretation of aspirin-based orthopaedic thromboprophylaxis. Nine RCTs, four major guideline documents, and sixteen additional Level I–II studies were included. Outcomes of interest were symptomatic deep vein thrombosis (DVT), pulmonary embolism (PE), major bleeding, and mortality. Risk of bias was assessed using the Cochrane ROB 2 framework. Owing to marked methodological heterogeneity, no formal pooled meta-analysis was undertaken. **Results:** The available RCT evidence suggests that aspirin may perform adequately within structured sequential or risk-stratified prophylaxis strategies, but not in all clinical settings. In arthroplasty, EPCAT II demonstrated non-inferiority of aspirin when introduced after an initial five-day course of rivaroxaban, whereas CRISTAL showed higher early symptomatic VTE rates when aspirin was used as sole primary prophylaxis from postoperative day 0. Importantly, thromboembolic events in CRISTAL occurred earlier in the aspirin cohort, supporting the concept that anticoagulant therapy remains important during the immediate postoperative hypercoagulable phase. In trauma surgery, PREVENT CLOT established non-inferiority of aspirin compared with LMWH for 90-day mortality; however, the predominantly young study population and the inclusion of upper-extremity fractures limit extrapolation to elderly hip fracture patients. Several smaller RCTs reported no major differences between aspirin and anticoagulants, but these studies were frequently underpowered and relied on less sensitive diagnostic strategies. Historical and contemporary guidelines remain heterogeneous, and evidence from modern fast-track arthroplasty pathways suggests that current trial-based conclusions may not be directly generalisable to short-duration prophylaxis settings. **Conclusions:** Aspirin may have a role in orthopaedic thromboprophylaxis when used within structured, risk-adapted or sequential protocols, particularly in standard-risk arthroplasty patients and selected trauma populations. However, current evidence does not support its universal use as sole primary prophylaxis in major orthopaedic surgery, especially during the early postoperative hypercoagulable phase or in high-risk patients. Furthermore, the available literature does not permit definitive recommendations regarding the optimal aspirin dose or duration of prophylaxis. The generalisability of the existing literature is further limited by methodological heterogeneity and by the absence of RCTs directly evaluating ultra-short anticoagulant regimens versus prolonged aspirin prophylaxis in modern fast-track arthroplasty. Further high-quality, standardised trials are required.

## 1. Introduction

Venous thromboembolism (VTE), encompassing deep vein thrombosis (DVT) and pulmonary embolism (PE), remains one of the most serious complications following major orthopaedic procedures such as total hip arthroplasty (THA), total knee arthroplasty (TKA), and fracture fixation [1]. Despite substantial advances in perioperative management, VTE continues to contribute to postoperative morbidity, mortality, prolonged hospitalisation, and increased healthcare costs [2]. Recent epidemiological data further underline the persistent global burden of postoperative thromboembolic disease despite contemporary preventive strategies [3]. Without pharmacological prophylaxis, reported rates of asymptomatic DVT following arthroplasty may approach 40–60% depending on diagnostic methodology, and symptomatic events remain clinically relevant in selected patient populations [4,5]. Enhanced Recovery After Surgery (ERAS) programmes emphasising early mobilisation, multimodal analgesia, and standardised perioperative protocols have substantially reduced thromboembolic risk, perioperative morbidity and shortened hospital stay. Modern fast-track arthroplasty pathways have additionally been associated with markedly lower contemporary symptomatic VTE rates than those historically reported in conventional arthroplasty cohorts [6,7,8]. Nevertheless, the optimal intensity and duration of pharmacological thromboprophylaxis within these pathways remain debated. Contemporary preventive strategies predominantly rely on low-molecular-weight heparins (LMWH), vitamin K antagonists, and direct oral anticoagulants (DOACs) [1]. While these agents demonstrate robust efficacy, they are associated with important limitations, including bleeding complications, wound haematoma, injection-related inconvenience, the need for laboratory monitoring, and higher direct costs [7,8,9].

Aspirin (acetylsalicylic acid, ASA) represents a low-cost, orally administered alternative with favourable safety characteristics. Through irreversible inhibition of platelet cyclooxygenase-1 and suppression of thromboxane A_2_ synthesis, aspirin primarily modulates platelet activation. Although venous thrombi are fibrin-rich, platelet activation contributes to thrombus propagation in the setting of surgical tissue injury, endothelial disruption, and inflammation. This provides a pathophysiological rationale for aspirin use in selected VTE prevention strategies. In addition, aspirin has become increasingly attractive within outpatient and accelerated recovery pathways because of its ease of administration and comparatively low bleeding risk.

The historical Pulmonary Embolism Prevention (PEP) trial demonstrated a 34% relative reduction in symptomatic VTE with low-dose aspirin in hip fracture and arthroplasty patients [5]. However, evolving surgical techniques and modern anticoagulants have reshaped interpretation of these findings. More recently, large randomised controlled trials such as EPCAT II [9], CRISTAL [10], PREVENT CLOT [11], and the risk-stratified trial by Kulshrestha et al., [12] have provided contemporary comparative data. Notably, EPCAT II evaluated sequential rivaroxaban–aspirin prophylaxis in standard-risk arthroplasty patients, whereas CRISTAL assessed aspirin initiated immediately postoperatively, demonstrating divergent early VTE rates. Importantly, CRISTAL reported earlier clustering of thromboembolic events in the aspirin group, suggesting that anticoagulant therapy may remain particularly important during the immediate postoperative hypercoagulable phase. Trauma-specific data further expand the discussion [11,13,14,15].

In parallel, multiple Bayesian network meta-analyses have consistently ranked aspirin as non-inferior to LMWH or DOACs in standard-risk arthroplasty populations [16,17,18,19,20]. However, these analyses aggregate heterogeneous trials with variable dosing regimens, diagnostic methodologies, and timing of prophylaxis initiation. Furthermore, perioperative factors such as tourniquet application [21], blood management protocols [22], fracture-related hypercoagulability [13,14,15], and laboratory markers of postoperative coagulation activation [23,24] highlight that thrombotic risk is highly procedure-specific and patient-dependent. Differences in endpoint definitions, particularly regarding asymptomatic DVT detection and major bleeding, further complicate direct comparisons across studies and meta-analyses.

Guideline recommendations reflect this complexity. The American Society of Hematology (ASH) provides a conditional recommendation for aspirin use in selected surgical patients [25]. NICE guidelines explicitly allow aspirin following TKA and sequentially after LMWH in THA [26]. The American College of Chest Physicians (ACCP) guidelines historically endorsed pharmacological prophylaxis after major orthopaedic surgery while acknowledging aspirin as a possible option in selected patients [27]. The American Academy of Orthopaedic Surgeons supports aspirin in typical-risk patients [28], whereas European perioperative guidelines emphasise individualised, risk-stratified prophylaxis strategies and acknowledge the evolving role of fast-track surgery pathways [29]. Such variability underscores ongoing uncertainty regarding optimal agent selection.

**Figure 1:**
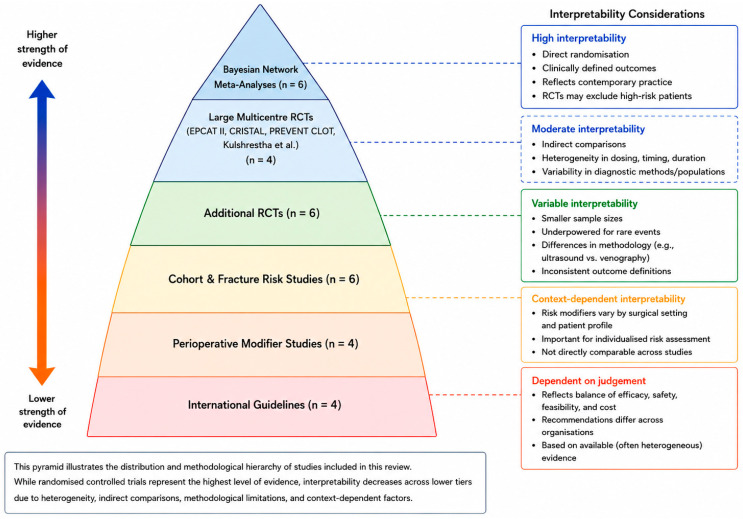
Hierarchy of Evidence Included in the Present Review. Legend: The pyramid illustrates the distribution and methodological hierarchy of studies included in this review. Bayesian network meta-analyses and large multicentre randomised controlled trials (RCTs) constitute the highest level of evidence, followed by additional RCTs, cohort and fracture-risk studies, perioperative modifier investigations, and international clinical practice guidelines.

Given the expanding and sometimes conflicting evidence base, a critical synthesis of contemporary RCTs, meta-analyses, and international guidelines is warranted. The aim of this review is therefore to summarise and contextualise current evidence regarding the role of aspirin in orthopaedic VTE prophylaxis. Particular emphasis is placed on risk stratification, timing of initiation, comparative safety, diagnostic methodology, and clinical applicability within contemporary arthroplasty and trauma pathways. The hierarchy of evidence included in this review is illustrated in Figure 1.

Given the heterogeneity in trial design, timing of prophylaxis initiation, diagnostic criteria, and patient risk profiles, a structured synthesis of contemporary RCTs and high-level meta-analytic data is necessary to clarify where aspirin provides genuine clinical equivalence and where anticoagulant therapy remains superior.

## 2. Methods

**Figure 2:**
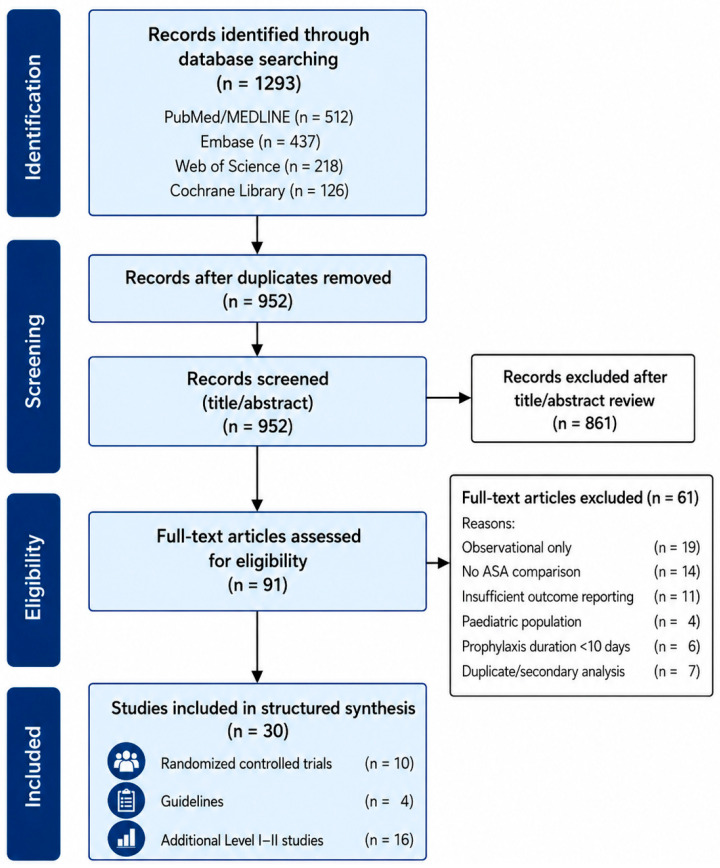
PRISMA 2020 flow diagram illustrating identification, screening, eligibility assessment, and inclusion of studies evaluating aspirin for venous thromboembolism prophylaxis following orthopaedic surgery.

This structured review was conducted in accordance with the Preferred Reporting Items for Systematic Reviews and Meta-Analyses (PRISMA 2020) statement (Figure 2). Because this review combined structured evidence synthesis with narrative contextualisation of perioperative modifiers and guideline recommendations, no prospective PROSPERO registration was performed. A comprehensive search of PubMed/MEDLINE, Embase, Web of Science, and the Cochrane Library was performed from database inception through September 2025. The following search terms and Boolean combinations were used: “aspirin” OR “acetylsalicylic acid” AND “venous thromboembolism” OR “deep vein thrombosis” OR “pulmonary embolism” AND “thromboprophylaxis” AND “arthroplasty” OR “total hip arthroplasty” OR “total knee arthroplasty” OR “fracture” OR “orthopaedic surgery”. The PRISMA 2020 checklist is provided as Supplementary Table S1. Database-specific search syntaxes, Boolean operators, MeSH/Emtree terms, and applied restrictions are provided in Supplementary Table S2.

### 2.1. Eligibility Criteria

Inclusion criteria:

- Randomised controlled trials (RCTs) comparing aspirin with LMWH, DOACs, vitamin K antagonists, or placebo;
- Adult patients (≥18 years) undergoing THA, TKA, fracture fixation, or other major orthopaedic procedures;
- Studies reporting symptomatic VTE, major bleeding, or mortality;
- Major international clinical practice guidelines addressing aspirin in orthopaedic VTE prophylaxis;
- High-level meta-analyses (Bayesian or network meta-analyses) published 2021–2025.

Exclusion criteria:

- Purely observational studies (except when addressing perioperative risk modifiers relevant to interpretation);
- Case reports or case series;
- Paediatric populations;
- Studies with prophylaxis duration <10 days;
- Studies lacking clear outcome definitions.

The restriction of meta-analyses to publications between 2021 and 2025 was chosen to reflect contemporary arthroplasty practice following the publication of EPCAT II and CRISTAL and to minimise incorporation of outdated prophylaxis pathways no longer representative of current perioperative care. Although the primary analysis focused on RCTs, selected high-quality multicentre cohort studies were included to contextualise fracture-related thrombotic risk and perioperative modifiers [13,14,15,16,17,18,19,20,21,22,23,24,25,26,27,28,29].

### 2.2. Study Selection

Two independent reviewers screened titles and abstracts for eligibility. Full-text assessment was performed for potentially relevant articles. Disagreements were resolved by consensus.

The complete study identification and selection process is illustrated in the PRISMA 2020 flow diagram (Figure 2).

### 2.3. Data Extraction

For each eligible study, the following data were extracted:

- Study design and setting
- Sample size
- Surgical population (THA, TKA, trauma, spine)
- Aspirin dosage and timing of initiation
- Comparator agent and dosing
- Duration of prophylaxis
- Diagnostic modality for VTE detection (venography, ultrasonography, symptomatic assessment)
- Definition of major bleeding
- Length of follow-up

Absolute event rates for symptomatic VTE and major bleeding were recorded when available.

Special attention was paid to methodological characteristics potentially influencing external validity, including patient risk stratification, fast-track perioperative pathways, timing of discharge, and whether thromboprophylaxis was initiated immediately postoperatively or sequentially after short-term anticoagulation.

### 2.4. Risk of Bias and Methodological Considerations

Particular attention was paid to methodological heterogeneity, including:

- Timing of prophylaxis initiation (day 0 vs. sequential strategy)
- Variability in aspirin dosing regimens (81–325 mg once or twice daily)
- Differences in DVT detection methods
- Exclusion of high-risk populations (e.g., prior VTE, active malignancy)
- Variability in definitions of “major bleeding”

**Figure 3:**
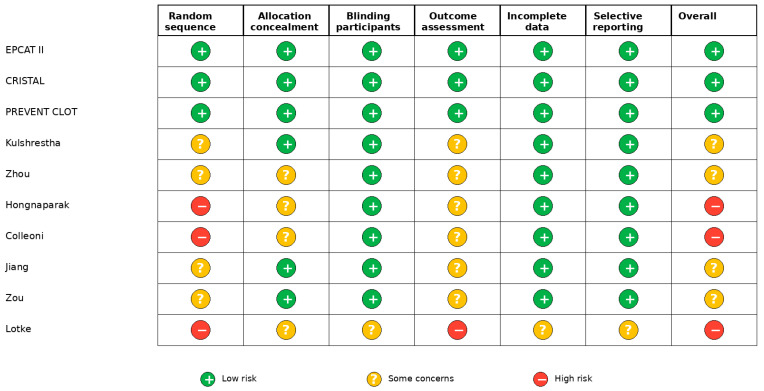
Study-level risk of bias assessment according to the Cochrane ROB 2 framework. Domains included randomisation process, deviations from intended interventions, missing outcome data, outcome measurement, and selection of reported results.

The risk of bias of included RCTs was assessed using the revised Cochrane Risk of Bias tool (ROB 2), including evaluation of the randomisation process, deviations from intended interventions, missing outcome data, outcome measurement, and selective reporting. Study-level ROB 2 assessments are presented in Figure 3.

Given the heterogeneity in study design and outcome definitions, diagnostic methodology, and perioperative protocols, no formal pooled meta-analysis was performed. Instead, findings were synthesised narratively, with emphasis on high-quality RCTs, guideline interpretation, and contemporary Bayesian network meta-analyses [16,17,18,19,20,30,31,32,33,34,35].

Because several smaller studies relied predominantly on ultrasonography rather than venography for DVT detection and were underpowered for symptomatic VTE endpoints, particular caution was exercised when interpreting apparent equivalence between aspirin and anticoagulant strategies.

## 3. Results

### 3.1. Overview of Included Evidence

A total of 1293 records were identified through database searching. Following duplicate removal, 952 records underwent title and abstract screening, of which 91 full-text articles were assessed for eligibility. Ultimately, 30 studies were included in the structured synthesis. Ten randomised controlled trials (RCTs), four major international guidelines, and sixteen additional Level I–II studies were included (Figure 2). The methodological quality assessment is presented in Figure 3. The detailed characteristics of the included RCTs are summarised in Table 1. The evidence base comprises large pragmatic multicentre trials, smaller single-centre RCTs, Bayesian network meta-analyses, and selected fracture-related cohort studies addressing perioperative thrombotic risk modifiers.

The included evidence demonstrated substantial heterogeneity regarding aspirin dosage (81–325 mg once or twice daily), timing of prophylaxis initiation, duration of treatment, diagnostic methodology for DVT detection, and definitions of major bleeding. These differences precluded formal pooled quantitative synthesis and necessitated narrative interpretation of the available evidence.

### 3.2. Arthroplasty: Large Randomised Trials

#### 3.2.1. EPCAT II (Sequential Prophylaxis)

The EPCAT II trial evaluated 3424 patients undergoing THA or TKA and demonstrated that, following five days of rivaroxaban, switching to aspirin 81 mg once daily was non-inferior to continued rivaroxaban for prevention of symptomatic VTE (0.64% vs. 0.70%) [9]. Importantly, patients at high thrombotic risk, including individuals with prior VTE, active malignancy, or known thrombophilia, were excluded from the study, thereby limiting extrapolation to higher-risk arthroplasty populations. Consequently, the findings primarily apply to standard-risk elective arthroplasty patients treated within structured sequential prophylaxis pathways.

#### 3.2.2. CRISTAL (Aspirin as Primary Prophylaxis)

In contrast, the CRISTAL trial randomised 9711 patients to aspirin 100 mg daily initiated on postoperative day 0 or enoxaparin 40 mg daily. Symptomatic VTE occurred significantly more frequently in the aspirin group (3.45% vs. 1.82%) [10]. Bleeding rates were comparable. Notably, thromboembolic events occurred earlier in the aspirin cohort (median approximately 7.5 days vs. 12 days), suggesting that immediate postoperative anticoagulation may provide superior protection during the early postoperative hypercoagulable phase. These findings support the concept that aspirin may be more effective as continuation prophylaxis following short-term anticoagulant administration rather than as sole immediate postoperative monotherapy.

These two large trials collectively suggest that aspirin performs best when used sequentially rather than as sole primary prophylaxis immediately after surgery. A structured overview of dosing strategies, comparators, and primary outcomes is provided in Table 1.

### 3.3. Trauma Population

The PREVENT CLOT trial included 12,211 patients with operatively treated extremity fractures or pelvic/acetabular trauma and compared aspirin 81 mg twice daily with enoxaparin 30 mg twice daily. Aspirin was non-inferior to LMWH with respect to 90-day mortality [11]. Rates of pulmonary embolism and major bleeding were similar, although a modest increase in distal DVT events was observed with aspirin.

It is noteworthy that the PREVENT CLOT population was relatively young (mean age approximately 45 years), limiting extrapolation to elderly hip fracture patients, who exhibit substantially higher baseline thrombotic risk [14,15]. Furthermore, approximately one-third of included injuries involved upper-extremity fractures, which may additionally reduce generalisability to frail geriatric hip fracture populations commonly encountered in routine orthopaedic practice.

Historical trauma evidence from the Pulmonary Embolism Prevention (PEP) trial demonstrated a relative reduction in symptomatic VTE with low-dose aspirin after hip fracture surgery [5]. However, interpretation of these findings remains controversial because the study preceded contemporary ERAS pathways, modern anticoagulants, and current perioperative thromboprophylaxis standards.

### 3.4. Risk-Stratified Strategy

Kulshrestha et al. randomised approximately 1000 TKA patients to routine anticoagulation or a risk-adapted strategy incorporating aspirin in low-risk individuals [12]. VTE incidence was comparable between groups, whereas bleeding complications were reduced in the risk-stratified cohort. This trial supports individualised prophylaxis based on baseline thrombotic risk and represents one of the few prospective evaluations of risk-adapted aspirin use in arthroplasty.

### 3.5. Smaller RCTs

Several smaller RCTs conducted in Asia and South America compared aspirin with rivaroxaban, LMWH, or warfarin regimens [30,31,32,33,34,35]. These trials, including studies by Zhou, Hongnaparak, Colleoni, Jiang, Zou, and Lotke, consistently reported no statistically significant differences in rates of DVT, PE, or major bleeding between aspirin (81–325 mg once or twice daily) and comparator anticoagulants.

However, these trials were limited by small sample size and frequent reliance on ultrasonography rather than venography for DVT detection. Because ultrasonography has substantially lower sensitivity than venography for asymptomatic distal DVT detection, these studies may have been underpowered to identify clinically relevant differences between aspirin and anticoagulant strategies. Consequently, their inferential strength remains limited.

In addition, substantial variability in aspirin dosing regimens prevents definitive conclusions regarding optimal aspirin dose or frequency. While lower doses such as 81 mg once daily may reduce gastrointestinal adverse effects, no trial has conclusively demonstrated superiority of one dosing strategy over another.

### 3.6. Bayesian Network Meta-Analyses (2021–2025)

Multiple contemporary Bayesian network meta-analyses comparing aspirin, LMWH, DOACs, and sequential regimens consistently ranked aspirin as non-inferior in preventing symptomatic VTE in standard-risk arthroplasty populations [16,17,18,19,20].

In pooled analyses, aspirin demonstrated among the lowest major bleeding rates. However, substantial heterogeneity was observed across trials regarding:

- Timing of prophylaxis initiation
- Aspirin dose (81–325 mg)
- Duration of therapy (2–6 weeks)
- DVT detection methodology
- Definitions of major bleeding

These methodological differences limit the precision of indirect comparisons. Furthermore, because several meta-analyses incorporated smaller underpowered studies and mixed symptomatic and asymptomatic endpoints, interpretation of pooled equivalence results requires caution.

### 3.7. Perioperative Risk Modifiers

Additional Level II studies highlighted important perioperative factors influencing thrombotic risk:

- Tourniquet use during TKA was associated with increased thromboembolic events in a Bayesian network meta-analysis [21].
- Sequential administration of tranexamic acid and haemocoagulase reduced blood loss without increasing VTE [22].
- In non-major orthopaedic surgery, rivaroxaban and LMWH demonstrated similar safety profiles [23].
- Multicentre fracture studies identified substantial preoperative DVT incidence in intertrochanteric and femoral shaft fractures [14,15].
- Thromboelastography studies revealed persistent postoperative hypercoagulability despite LMWH prophylaxis [24].

These findings underscore that VTE risk is highly dependent on surgical procedure, tissue trauma, and patient-specific factors.

At the same time, modern fast-track arthroplasty cohorts have reported substantially lower symptomatic VTE rates using accelerated mobilisation and abbreviated thromboprophylaxis protocols. This discrepancy highlights that many historical RCTs may not fully reflect current ERAS-based arthroplasty practice.

### 3.8. Guideline Recommendations

Four major guideline bodies were identified:

- The American Society of Hematology suggests aspirin or anticoagulants for THA/TKA with low-certainty evidence [25].
- NICE permits aspirin after TKA or sequentially following LMWH in THA [26].
- The American Academy of Orthopaedic Surgeons endorses aspirin (81–325 mg twice daily for 4–6 weeks) in typical-risk patients [28].
- The European Society of Cardiology emphasises individualised, risk-stratified prophylaxis [29].

Additionally, the American College of Chest Physicians (ACCP) guidelines historically recognised aspirin as a possible thromboprophylactic option after major orthopaedic surgery, although stronger recommendations were generally reserved for anticoagulant strategies [27].

The variability among guideline recommendations reflects the heterogeneity of the underlying evidence base, differing interpretation of the CRISTAL and EPCAT II findings, variation in accepted baseline risk thresholds, and evolving perspectives regarding fast-track arthroplasty pathways and abbreviated prophylaxis strategies.

## 4. Discussion

### 4.1. Principal Findings

The present review synthesises evidence from ten randomised controlled trials, four international guidelines, and sixteen additional Level I–II studies to clarify the role of aspirin (ASA) in venous thromboembolism (VTE) prophylaxis following orthopaedic surgery. Collectively, the data indicate that aspirin represents a viable component of thromboprophylaxis strategies; however, its efficacy and appropriateness are strongly influenced by patient-specific risk profile, surgical context, timing of initiation, and duration of prophylaxis.

Importantly, the currently available evidence primarily reflects standard-risk arthroplasty populations and selected trauma cohorts. High-risk patients, including individuals with prior VTE, active malignancy, thrombophilia, severe obesity, or prolonged immobilisation, remain underrepresented in the contemporary RCT literature.

### 4.2. Arthroplasty: Sequential Versus Primary Prophylaxis

The most robust evidence originates from total hip and knee arthroplasty populations. The EPCAT II trial demonstrated that switching to aspirin after a brief five-day course of rivaroxaban resulted in VTE rates indistinguishable from continued rivaroxaban therapy (0.64% vs. 0.70%) [9]. This finding established aspirin as an effective continuation strategy in predominantly standard-risk arthroplasty patients.

In contrast, the CRISTAL trial revealed significantly higher symptomatic VTE rates when aspirin 100 mg daily was initiated as sole prophylaxis from postoperative day 0 (3.45% vs. 1.82% with enoxaparin) [10]. Importantly, bleeding outcomes were similar between groups. Furthermore, thromboembolic events occurred earlier in the aspirin arm, reinforcing the concept that anticoagulant therapy may provide superior protection during the immediate postoperative hypercoagulable phase. These findings collectively suggest that aspirin appears most reliable as extended prophylaxis following initial anticoagulant administration rather than as sole primary prophylaxis immediately after major arthroplasty.

From a pathophysiological perspective, major orthopaedic surgery induces substantial thrombin generation and activation of the coagulation cascade. Aspirin, as a platelet inhibitor, does not directly suppress factor Xa or thrombin activity, which may explain reduced early efficacy compared with LMWH or DOACs.

At the same time, interpretation of historical arthroplasty RCTs must consider the rapid evolution of modern ERAS and fast-track surgery pathways. Contemporary fast-track arthroplasty cohorts have demonstrated markedly lower symptomatic VTE rates using accelerated mobilisation and abbreviated thromboprophylaxis protocols than those historically reported in conventional arthroplasty populations [6,7,8]. Consequently, direct extrapolation of older RCTs to current short-stay arthroplasty practice may be limited.

### 4.3. THA Versus TKA: Distinct Clinical Risk Profiles

Importantly, total hip arthroplasty (THA) and total knee arthroplasty (TKA) should not be regarded as interchangeable thromboembolic risk models. Contemporary THA pathways have progressively evolved toward enhanced recovery protocols characterised by early mobilisation, shortened hospital stay, multimodal analgesia, and optimised blood-management strategies. These developments may have substantially altered the baseline VTE risk profile compared with historical arthroplasty cohorts and may partly explain the favourable outcomes observed in some contemporary aspirin-based strategies [36].

In contrast, TKA is associated with several procedure-specific factors that may influence postoperative thrombosis risk, including tourniquet use, greater local inflammatory activation, postoperative pain-related mobilisation delay, and distinct patterns of coagulation activation [20,37]. Consequently, findings derived from mixed THA/TKA populations should be interpreted cautiously, as prophylaxis strategies that appear adequate in standard-risk fast-track THA populations may not necessarily provide equivalent protection in TKA or more complex revision settings.

These considerations further support the growing trend toward individualised thromboprophylaxis strategies that integrate patient frailty, bleeding risk, mobilisation capacity, procedural characteristics, and baseline thromboembolic risk rather than relying on a uniform arthroplasty-wide approach.

### 4.4. Trauma and Fracture Surgery

The PREVENT CLOT trial provided large-scale evidence in trauma populations, demonstrating non-inferiority of aspirin compared with LMWH with respect to 90-day mortality in over 12,000 patients [11]. Pulmonary embolism and major bleeding rates were comparable, although distal DVT occurred slightly more frequently in the aspirin group.

However, the PREVENT CLOT cohort was relatively young (mean age approximately 45 years), limiting extrapolation to elderly hip fracture populations, who exhibit higher thrombotic burden and perioperative immobility [14,15]. Additionally, approximately one-third of included injuries involved upper-extremity fractures, which likely reduced overall thrombotic event rates and further limits applicability to frail geriatric fracture patients.

Additional multicentre fracture studies have demonstrated significant baseline DVT incidence in intertrochanteric and femoral shaft fractures [14,15], emphasising the importance of risk stratification in this subgroup.

Historical evidence from the Pulmonary Embolism Prevention (PEP) trial demonstrated reduced symptomatic VTE with low-dose aspirin following hip fracture surgery [5]. However, this study preceded current ERAS concepts, contemporary anticoagulants, and modern thromboprophylaxis protocols, limiting direct comparability to current orthopaedic practice.

### 4.5. Smaller RCTs and Real-World Heterogeneity

Several smaller trials conducted in Asia and South America compared aspirin with rivaroxaban, warfarin, or sequential LMWH regimens [30,31,32,33,34,35]. Although these trials were underpowered individually, they consistently reported no statistically significant differences in VTE or major bleeding between aspirin and anticoagulants.

Notably, these studies employed diverse aspirin doses (81–325 mg once or twice daily), prophylaxis durations (2–6 weeks), and adjunctive measures such as mechanical prophylaxis. This heterogeneity reflects real-world practice variability and may enhance external validity, yet it substantially limits direct comparability and precludes definitive conclusions regarding optimal aspirin dose or duration.

Diagnostic heterogeneity further complicates interpretation. Some trials relied on routine ultrasonography, whereas others reported only symptomatic events. Because ultrasonography has substantially lower sensitivity than venography for asymptomatic distal DVT detection, several smaller studies may have underestimated true event rates and biased results toward apparent equivalence between aspirin and anticoagulant strategies.

### 4.6. Meta-Analytic Evidence and Its Limitations

Multiple Bayesian network meta-analyses (2021–2025) have consistently ranked aspirin as non-inferior to LMWH and DOACs for prevention of symptomatic VTE in standard-risk arthroplasty populations [16,17,18,19,20]. In pooled analyses, aspirin frequently demonstrated among the lowest major bleeding rates.

However, network meta-analyses inherently depend on indirect comparisons across trials with heterogeneous inclusion criteria, dosing regimens, outcome definitions, and perioperative protocols. Variability in timing of prophylaxis initiation—particularly sequential versus immediate postoperative aspirin—may significantly confound pooled effect estimates.

Moreover, several meta-analyses incorporated smaller underpowered studies using less sensitive ultrasonographic DVT detection methods and mixed symptomatic with asymptomatic endpoints. Consequently, formal Level I classification of meta-analytic evidence should be interpreted cautiously in the setting of marked orthopaedic methodological heterogeneity.

### 4.7. Risk Stratification and Procedure-Specific Considerations

Emerging data highlight the importance of perioperative risk modifiers. Tourniquet use during TKA has been associated with increased thromboembolic risk in a Bayesian meta-analysis [21]. Blood management strategies, including sequential tranexamic acid and haemocoagulase administration, may reduce bleeding without increasing VTE risk [22]. Laboratory studies demonstrate persistent hypercoagulability following arthroplasty despite LMWH prophylaxis [24], reinforcing the biological plausibility of early anticoagulant therapy prior to transition to aspirin.

These findings support a procedure-specific and risk-adapted prophylaxis model rather than uniform application of aspirin across all surgical contexts. At present, however, no universally accepted risk-stratification model adequately integrates perioperative modifiers such as hypercoagulability profiles, tourniquet use, or ERAS pathway variables into routine thromboprophylaxis algorithms.

### 4.8. Safety Considerations

Across included RCTs, aspirin did not increase major bleeding, wound complications, or mortality compared with anticoagulants. Observational and pooled analyses suggest lower bleeding rates with aspirin relative to DOACs [16,17,18,19,20].

Nevertheless, definitions of “major bleeding” varied considerably across trials. Some definitions excluded clinically relevant wound haematoma or prolonged drainage, which may underestimate true surgical morbidity. Furthermore, bleeding ascertainment methods differed substantially between studies and even within individual drug development programmes. Caution is therefore warranted when interpreting safety superiority.

### 4.9. Guideline Interpretation

Guideline recommendations remain heterogeneous. The American Society of Hematology provides a conditional recommendation for aspirin use in selected surgical patients [25]. NICE permits aspirin monotherapy after TKA and sequential use following LMWH in THA [26]. The American Academy of Orthopaedic Surgeons endorses aspirin for typical-risk patients (81–325 mg twice daily for 4–6 weeks) [28], whereas the European Society of Cardiology emphasises individualised, risk-stratified prophylaxis [29]. The American College of Chest Physicians (ACCP) guidelines additionally recognised aspirin as a possible thromboprophylactic option after major orthopaedic surgery, although anticoagulant strategies generally received stronger recommendations [27].

These discrepancies likely reflect differing emphasis on efficacy versus safety, cost considerations, interpretation of CRISTAL versus EPCAT II, accepted baseline risk thresholds, and evolving perspectives regarding ERAS and fast-track arthroplasty pathways.

### 4.10. Strengths and Limitations

The strengths of the current synthesis include inclusion of large pragmatic RCTs (EPCAT II, CRISTAL, PREVENT CLOT), contemporary Bayesian analyses, and integration of perioperative risk modifier studies. The methodological characteristics of the included trials are summarised in Table 1, and study-level ROB 2 assessments are illustrated in Figure 3.

Limitations include:

- Heterogeneity in comparator regimens
- Variable initiation timing
- Differences in prophylaxis duration
- Differences in diagnostic methodology
- Variable bleeding definitions
- Exclusion of high-risk populations (prior VTE, malignancy, severe obesity)
- Limited blinding in some RCTs

As illustrated in Figure 3, most methodological concerns related to blinding and outcome assessment in smaller single-centre trials. Consequently, generalisability to high-risk surgical patients remains uncertain.

### 4.11. Clinical Implications

The accumulated evidence supports a risk-stratified strategy:

- Sequential prophylaxis (initial anticoagulation followed by aspirin) in standard-risk arthroplasty patients
- Aspirin as a pragmatic alternative to LMWH in selected trauma populations
- Avoidance of aspirin monotherapy initiated immediately postoperatively in high-risk arthroplasty patients

Such an approach balances efficacy, safety, cost, and feasibility. However, evidence remains insufficient to recommend aspirin as universal sole primary prophylaxis across all major orthopaedic procedures or within all modern fast-track arthroplasty settings. From a practical perspective, the available evidence suggests that aspirin-based prophylaxis should be interpreted differently in THA and TKA populations. Modern fast-track THA pathways may permit broader implementation of risk-adapted aspirin strategies, whereas TKA patients often present procedure-specific factors warranting more cautious evaluation and individualised decision-making [36,37].

### 4.12. Future Directions

Future research should:

- Define optimal aspirin dosing (81 vs. 325 mg; once vs. twice daily)
- Determine ideal prophylaxis duration across surgical subgroups
- Directly compare ultra-short anticoagulant regimens versus prolonged aspirin strategies in modern fast-track arthroplasty
- Include high-risk and elderly fracture populations in adequately powered RCTs
- Standardise diagnostic and bleeding definitions to improve comparability
- Develop validated risk-stratification models integrating perioperative and patient-specific thrombotic modifiers

Taken together, the synthesis of trial data, meta-analytic evidence, perioperative risk modifiers, and guideline recommendations provides a structured framework for contextualising aspirin within contemporary orthopaedic thromboprophylaxis.

## 5. Conclusions

This comprehensive synthesis of randomised trials, contemporary Bayesian meta-analyses, perioperative risk-modifier studies, and international guidelines delineates a context-dependent role for aspirin (ASA) in orthopaedic venous thromboembolism (VTE) prophylaxis.

The accumulated evidence does not support aspirin as universal primary monotherapy initiated immediately after major arthroplasty. The CRISTAL trial demonstrated increased symptomatic VTE when aspirin was used from postoperative day 0 compared with enoxaparin [10], suggesting that anticoagulant therapy remains essential during the early postoperative hypercoagulable phase. Importantly, thromboembolic events occurred earlier in the aspirin arm, further supporting the concept that early postoperative anticoagulation may provide superior protection during the period of peak coagulation activation. Pathophysiological data and thromboelastography studies further corroborate sustained postoperative coagulation activation despite standard prophylaxis [24].

Conversely, the EPCAT II trial established that sequential prophylaxis—initial anticoagulation followed by aspirin—yields outcomes non-inferior to extended rivaroxaban in standard-risk arthroplasty patients [9]. This finding, supported by multiple Bayesian network meta-analyses [16,17,18,19,20], positions aspirin as a reliable continuation strategy rather than a stand-alone early intervention.

In trauma populations, PREVENT CLOT demonstrated non-inferiority of aspirin compared with LMWH with respect to 90-day mortality [11]. These results support aspirin as a pragmatic and scalable option in selected fracture patients. However, elderly hip fracture cohorts—characterised by higher baseline thrombotic risk [14,15]—may require more cautious implementation and further targeted investigation. In addition, the relatively young PREVENT CLOT study population and inclusion of upper-extremity fractures limit direct extrapolation to frail geriatric trauma populations.

Smaller RCTs across diverse healthcare systems consistently reported comparable efficacy between aspirin and rivaroxaban, warfarin, or sequential LMWH regimens [30,31,32,33,34,35]. Although individually underpowered, their convergence strengthens external validity. Importantly, substantial heterogeneity in dosing, timing, and diagnostic methodology across trials underscores that aspirin’s performance cannot be interpreted independently of clinical context.

Guideline recommendations remain heterogeneous, reflecting differing prioritisation of efficacy, safety, cost, and feasibility. While ASH offers a conditional endorsement [25], NICE and AAOS explicitly support aspirin in selected or typical-risk populations [26,28], and European guidance emphasises risk-stratified strategies [29]. The ACCP guidelines additionally recognise aspirin as a potential option in selected orthopaedic patients, although anticoagulant strategies generally retain stronger recommendations [27]. This variability mirrors the nuanced evidence base.

Taken together, aspirin appears most appropriately deployed within risk-adapted protocols. However, the optimal prophylaxis strategy may differ between contemporary THA and TKA pathways because of important differences in thrombotic risk, perioperative management, inflammatory response, mobilisation patterns, and recovery trajectories [36,37]:

- As extended prophylaxis following short-term anticoagulation in standard-risk arthroplasty;
- As a pragmatic alternative to LMWH in selected trauma patients;
- Avoided as sole primary prophylaxis immediately postoperatively in high-risk surgical contexts.

Its low cost, oral administration, and favourable bleeding profile make it particularly attractive in outpatient pathways and resource-constrained environments. However, early postoperative anticoagulation remains critical in patients with elevated thrombotic risk. Importantly, the currently available literature does not permit definitive conclusions regarding the optimal aspirin dose, dosing frequency, or ideal duration of prophylaxis. Likewise, modern fast-track arthroplasty pathways using ultra-short anticoagulant regimens remain insufficiently represented in current RCT evidence [6,7,8].

Future research should prioritise the following:

- Prospective trials separately evaluating contemporary THA and TKA populations, particularly elderly and high-risk patients;
- Direct comparison of ultra-short anticoagulant regimens versus prolonged aspirin strategies in modern ERAS arthroplasty Standardisation of bleeding and diagnostic definitions;
- Direct comparisons of dosing strategies (81 vs. 325 mg; once vs. twice daily);
- Development of validated risk-stratification algorithms integrating perioperative modifiers and patient-specific thrombotic risk.

In conclusion, aspirin is neither universally superior nor inferior to anticoagulants; rather, its optimal value lies within structured, risk-stratified thromboprophylaxis pathways tailored to the surgical setting and patient profile.

## Figures and Tables

**Table 1:** Characteristics of included randomised controlled trials.

Study	Population	ASA Regimen	Comparator	Duration	Primary Outcome
EPCAT II [9]	3424 THA/TKA	Rivaroxaban 5d → ASA 81 mg OD	Rivaroxaban	90 d	Symptomatic VTE
CRISTAL [10]	9711 THA/TKA	ASA 100 mg OD (day 0)	Enoxaparin 40 mg OD	90 d	Symptomatic VTE
PREVENT CLOT [11]	12,211 fractures	ASA 81 mg BID	Enoxaparin 30 mg BID	90 d	Mortality
Kulshrestha [12]	~1000 TKA	Risk-stratified ASA	Routine anticoagulation	14 d	VTE
Zhou [30]	120 TKA	ASA 100 mg OD	Rivaroxaban	90 d	VTE
Hongnaparak [31]	40 TKA	ASA 300 mg OD	Rivaroxaban	14 d	DVT
Colleoni [32]	27 TKA	ASA 150 mg BID	Rivaroxaban	4 w	VTE
Jiang [33]	120 TKA	ASA + mechanical	LMWH → DOAC	6 w	VTE
Zou [34]	212 TKA	ASA 100 mg OD	Rivaroxaban	4 w	VTE
Lotke [35]	192 THA/TKA	ASA 325 mg BID	Warfarin	6 m	VTE

## Data Availability

The data presented in this study are available from the corresponding author upon reasonable request. The data are not publicly available because the study is based on published literature and the compiled extraction files used for the review are maintained by the authors.

## Supplementary Materials

The following supporting information can be downloaded at https://www.mdpi.com/article/10.3390/jcm15124550/s1: Table S1: PRISMA 2020 Checklist; Table S2: Detailed Search Strategy.

## Abbreviations

AAOS	American Academy of Orthopaedic Surgeons
ASA	Acetylsalicylic acid (aspirin)
ASH	American Society of Hematology
CRISTAL	Comparison of Risk of Symptomatic Thromboembolism After Knee or Hip Arthroplasty Using Aspirin or Low-Molecular-Weight Heparin Trial
DVT	Deep vein thrombosis
DOAC	Direct oral anticoagulant
ERAS	Enhanced recovery after surgery
EPCAT II	Extended Prophylaxis Comparing Aspirin and Rivaroxaban II Trial
LMWH	Low-molecular-weight heparin
NICE	National Institute for Health and Care Excellence
PE	Pulmonary embolism
PRISMA	Preferred Reporting Items for Systematic Reviews and Meta-Analyses
RCT	Randomised controlled trial
THA	Total hip arthroplasty
TKA	Total knee arthroplasty
VTE	Venous thromboembolism
