# Supplementary material for: Aspirin for Venous Thromboembolism Prevention in Orthopaedic Surgery with Focus on Trauma and Arthroplasty: A Structured Evidence-Based Review of Randomised Trials, Guidelines, and Contemporary Practice Considerations
Source: J Clin Med. 2026 Jun 11;15(12):4550. doi: 10.3390/jcm15124550 (PMC13301266; doi:10.3390/jcm15124550)
Supplement: Supplementary file 1 [file jcm-15-04550-s001.zip › jcm-4311303-supplementary/Supplementary Table S1_PRISMA_2020_Checklist.pdf]

PRISMA 2020 CHECKLIST

Manuscript title:

Aspirin for Venous Thromboembolism Prevention in Orthopaedic Surgery with Focus on Trauma and Arthroplasty: A Structured Evidence-Based Review of Randomised Trials and Guidelines

Title

Item	Checklist item	Reported	Location
1	Identify the report as a systematic review	Yes	Title

Abstract

Item	Checklist item	Reported	Location
2	Structured abstract	Yes	Abstract

Introduction

Item	Checklist item	Reported	Location
3	Rationale	Yes	Introduction
4	Objectives	Yes	End of Introduction

Methods

Item	Checklist item	Reported	Location
5	Eligibility criteria	Yes	Methods
6	Information sources	Yes	Methods
7	Search strategy	Yes (FULLY)	Methods + Supplement Table S1
8	Selection process	Yes	Methods
9	Data collection process	Yes	Methods
10	Data items	Yes	Methods
11	Risk of bias assessment	Yes	Methods + Figure 3
12	Effect measures	Not applicable	Qualitative synthesis
13	Synthesis methods	Yes	Methods
14	Reporting bias assessment	Not performed	Stated in Methods
15	Certainty assessment	Not performed	Stated in Methods

Results

Item	Checklist item	Reported	Location
16	Study selection	Yes	Results + Figure 2
17	Study characteristics	Yes	Table 1

Item	Checklist item	Reported	Location
18	Risk of bias in studies	Yes	Figure 3
19	Results of individual studies	Yes	Results
20	Results of syntheses	Yes (qualitative)	Results
21	Reporting biases	Not assessed	Mentioned in Discussion
22	Certainty of evidence	Not assessed	Mentioned in Discussion

Discussion

Item	Checklist item	Reported	Location
23	Interpretation of results	Yes	Discussion
24	Limitations of evidence	Yes	Discussion
25	Limitations of review process	Yes	Discussion
26	Implications for practice/research	Yes	Discussion

Other Informations

Item	Checklist item	Reported	Location
27	Registration	Not registered	Not applicable
28	Protocol	Not available	Not applicable
29	Funding	Yes	Declarations
30	Competing interests	Yes	Declarations
31	Availability of data	Yes	Declarations
