# Supplementary material for: Aspirin for Venous Thromboembolism Prevention in Orthopaedic Surgery with Focus on Trauma and Arthroplasty: A Structured Evidence-Based Review of Randomised Trials, Guidelines, and Contemporary Practice Considerations
Source: J Clin Med. 2026 Jun 11;15(12):4550. doi: 10.3390/jcm15124550 (PMC13301266; doi:10.3390/jcm15124550)
Supplement: Supplementary file 1 [file jcm-15-04550-s001.zip › jcm-4311303-supplementary/Supplementary Table S2_Detailed Search Strategy.pdf]

Supplementary Table S2. Detailed Search Strategy

Database	Search Strategy	Filters	Date
**PubMed (MEDLINE)**	("aspirin"[Title/Abstract] OR "acetylsalicylic acid"[Title/Abstract]) AND ("venous thromboembolism"[Title/Abstract] OR "VTE"[Title/Abstract] OR "deep vein thrombosis"[Title/Abstract] OR "DVT"[Title/Abstract] OR "pulmonary embolism"[Title/Abstract] OR "PE"[Title/Abstract]) AND ("orthopedic surgery"[Title/Abstract] OR "orthopaedic surgery"[Title/Abstract] OR "arthroplasty"[Title/Abstract] OR "joint replacement"[Title/Abstract] OR "hip arthroplasty"[Title/Abstract] OR "knee arthroplasty"[Title/Abstract] OR "fracture"[Title/Abstract] OR "trauma"[Title/Abstract]) AND ("randomized controlled trial"[Publication Type] OR "RCT"[Title/Abstract])	Humans, Adults, English	September 2025
**Embase**	('aspirin'/exp OR 'acetylsalicylic acid') AND ('venous thromboembolism'/exp OR 'deep vein thrombosis' OR 'pulmonary embolism') AND ('orthopedic surgery'/exp OR 'arthroplasty' OR 'joint replacement' OR 'fracture' OR 'trauma') AND ('randomized controlled trial'/exp)	Humans, Adults, English	September 2025
**Web of Science**	TS=("aspirin" OR "acetylsalicylic acid") AND TS=("venous thromboembolism" OR "deep vein thrombosis" OR "pulmonary embolism") AND TS=("arthroplasty" OR "orthopedic surgery" OR "fracture" OR "trauma") AND TS=("randomized controlled trial")	English	September 2025
**Cochrane Library**	("aspirin" OR "acetylsalicylic acid") AND ("venous thromboembolism" OR "deep vein thrombosis" OR "pulmonary embolism") AND ("arthroplasty" OR "fracture" OR "orthopedic surgery")	Trials	September 2025

Abbreviations: VTE, venous thromboembolism; DVT, deep vein thrombosis; PE, pulmonary embolism; RCT, randomized controlled trial.
